# The HDAC–Lactylation Axis in Cancer Chemoresistance: A Metabolic–Epigenetic Interface

**DOI:** 10.3390/ijms272110000

**Published:** 2026-11-08

**Authors:** Nhi Thi Tuyet Hoang, So-Jin Shin, Jin Young Kim, Ji Hae Seo

**Affiliations:** 1 Department of Biochemistry, School of Medicine, Keimyung University, Daegu 42601, Republic of Korea; tuyetnhi2907@gmail.com; 2 Department of Gynecology and Obstetrics, School of Medicine, Keimyung University, Daegu 42601, Republic of Korea; hope2014@dsmc.or.kr; 3 Department of Internal Medicine, School of Medicine, Keimyung University, Daegu 42601, Republic of Korea

**Keywords:** lactylation, histone deacetylases (HDACs), chemoresistance, cancer metabolism, epigenetic plasticity

## Abstract

Chemoresistance remains a critical obstacle in cancer therapy, shaped not only by genetic mutations but also by reversible metabolic and epigenetic adaptations. Enhanced glycolysis and lactate accumulation are associated with tumor fitness before and during therapy and position lactate as more than a metabolic byproduct, enabling it to act as a regulatory metabolite through lysine lactylation of histone and non-histone proteins. This review examines the HDAC–lactylation axis as a metabolic–epigenetic interface and asks whether HDAC-mediated regulation of lactylation causally influences chemotherapy resistance or sensitization. We summarize evidence on lactate production, protein lactylation in cancer, and class I histone deacetylases (HDAC1–3) as delactylases, highlighting the reversible nature of this modification. Current evidence implicates histone and non-histone lactylation in two processes relevant to chemotherapy response: histone lactylation-dependent transcriptional programs involved in metabolic stress responses and phenotypic plasticity, and direct regulation of non-histone proteins involved in DNA repair, RNA processing, genome stability, and cellular stress adaptation. Together, these findings suggest that the HDAC–lactylation axis functions as a dynamic metabolic–epigenetic regulatory network enabling context-dependent adaptive responses. HDAC isoform-focused pharmacological and genetic perturbation, inhibition of lactate dehydrogenase A (LDHA) or monocarboxylate transporter 1 (MCT1), suppression of monocarboxylate transporter 4 (MCT4), and site-specific lactylation proteomics using liquid chromatography–tandem mass spectrometry (LC-MS/MS) could clarify the contribution of this axis to chemotherapy response.

## 1. Introduction

Chemoresistance remains a major obstacle in cancer therapy and is increasingly recognized as a multifactorial process that extends beyond genetic mutations. In addition to stable genomic alterations, cancer cells can adopt reversible adaptive states that enable survival under therapeutic stress, highlighting the importance of non-genetic mechanisms in treatment failure [1,2,3]. Among these, epigenetic regulation has emerged as a central contributor to transcriptional plasticity, allowing dynamic reprogramming of gene expression without permanent changes to DNA sequence [4]. In parallel, tumor metabolism has been extensively studied, particularly the role of aerobic glycolysis (the Warburg effect) in supporting cancer progression [5].

Lactate, long considered a metabolic byproduct, is now understood to function as a signaling molecule that influences multiple aspects of tumor biology, including immune evasion, angiogenesis, and therapeutic resistance [6]. More recently, the discovery of histone lysine lactylation has provided a direct molecular link between metabolic rewiring and gene regulation [7]. This modification reflects intracellular lactate availability and has been associated with activation of transcriptional programs involved in tumor progression and adaptation [7,8,9]. Importantly, lactylation is not a static modification. Its dynamic and reversible nature implies the existence of regulatory enzymes that control its turnover [10,11,12]. Lactylation can be established through enzymatic mechanisms involving lactyl-group transfer. The acetyltransferase p300 can catalyze histone lactylation [7,13], while ACSS2 generates lactyl-CoA for KAT2A-mediated histone lactylation [14]. AARS1 can mediate lactylation through a lactyl-AMP intermediate without requiring lactyl-CoA [15]. In this context, class I histone deacetylases (HDAC1–3) have been identified as histone lysine delactylases, demonstrating that lactylation can be actively removed and modulated [12]. NAD^+^-dependent sirtuins, including SIRT1–3 and SIRT6, also exhibit delactylase activity [12,16,17]. Accordingly, this review focuses primarily on HDAC1–3 because of their reported involvement in lactylation-associated mechanisms relevant to chemotherapy response, while acknowledging the broader contributions of sirtuins.

From a conceptual perspective, these observations suggest that lactate-driven lactylation and HDAC-mediated delactylation together form a reversible regulatory system linking metabolic state to transcriptional and protein-level regulation. This system operates as a dynamic interface through which fluctuations in cellular metabolism can be translated into epigenetic and transcriptional changes [7,10,11,12]. Such reversibility is particularly relevant in cancer, where adaptive responses to therapy often depend on transient and plastic cellular states [1,2,3]. In addition, emerging evidence indicates that lactylation is not restricted to histones but can also occur on non-histone proteins, thereby extending its regulatory impact beyond chromatin to the direct modulation of protein activity, stability, and molecular interactions [16,17,18,19]. These findings suggest that metabolite-derived modifications can affect both chromatin-based gene regulation and the post-translational control of protein function [16,17,20]. In selected experimental models, HDAC-associated changes in histone and non-histone lactylation have been linked to chemotherapy resistance or sensitization, although their functional consequences depend on the modified substrate and therapeutic context [19,20,21].

**Figure 1:**
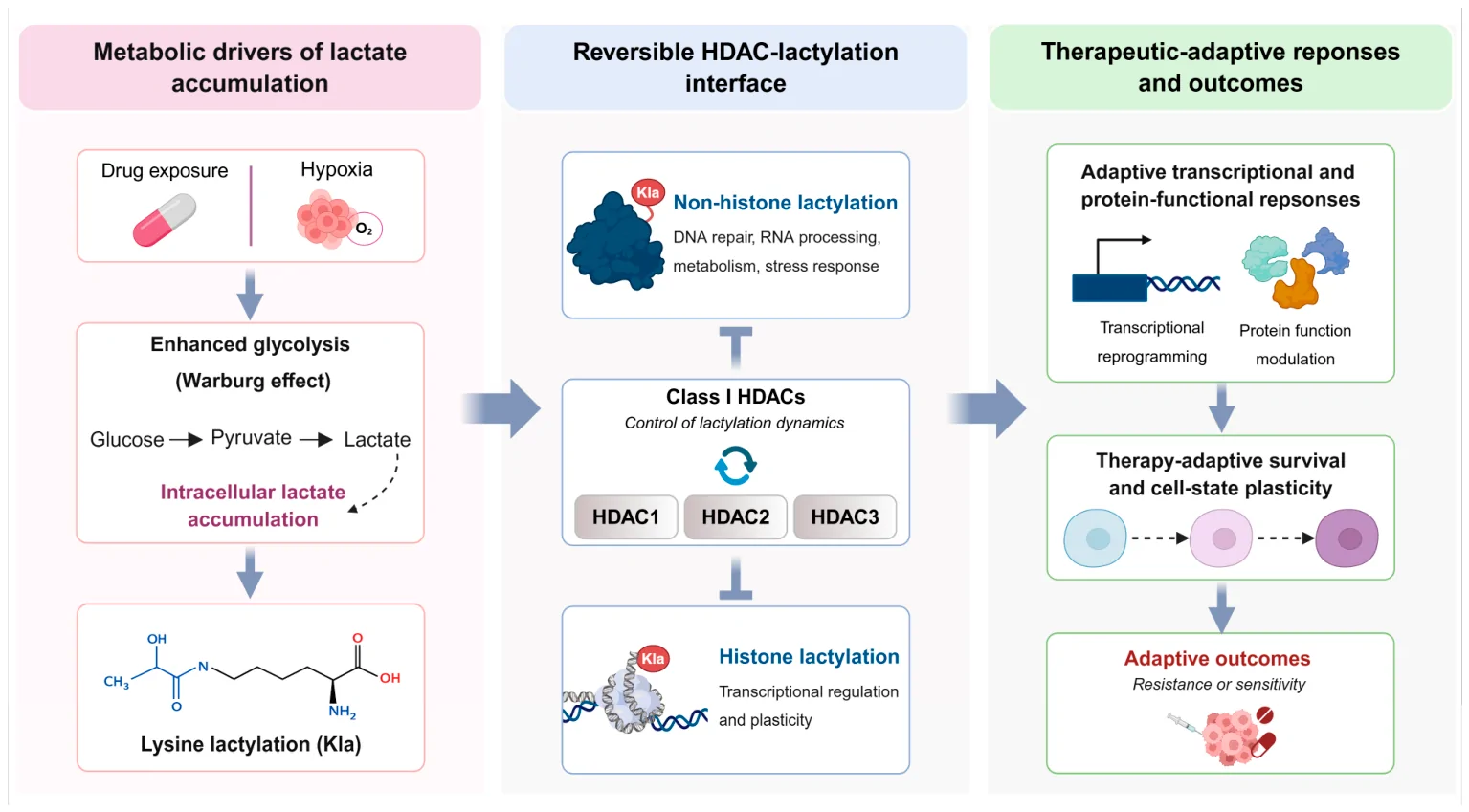
The HDAC–lactylation axis as a metabolic–epigenetic interface in cancer chemoresistance. Tumor glycolysis-driven lactate accumulation promotes histone and non-histone lactylation. HDAC1–3 dynamically regulate lactylation turnover, linking metabolic state to adaptive transcription and protein function. These reversible responses support therapy-adaptive survival and contribute to chemoresistance. Abbreviations: HDAC, histone deacetylase; Kla, lysine lactylation; DNA, deoxyribonucleic acid; RNA, ribonucleic acid. (Created in BioRender. Nguyen, E. (2026) https://BioRender.com/wiq5ppj, accessed on 4 November 2026).

Based on these developments, this review focuses on the HDAC–lactylation axis as a metabolic–epigenetic framework linking transcriptional plasticity and protein-level regulation to adaptive responses in cancer. Although lactylation also contributes to cancer-associated processes outside chemotherapy, treatment response provides a clinically relevant context for evaluating whether these regulatory mechanisms influence resistance or sensitization. Specifically, this narrative review examines whether, and under what molecular and therapeutic conditions, HDAC-mediated regulation of lactylation causally influences chemotherapy response, with particular emphasis on mechanistic evidence from experimental models of solid tumors. The discussion prioritizes studies that directly evaluate chemotherapy response in relation to defined lactylation sites, their regulatory enzymes, and downstream molecular pathways. Studies conducted outside chemotherapy settings are included when they provide essential biochemical or mechanistic context but are distinguished from direct evidence of chemotherapy resistance or sensitization. By integrating current evidence on chromatin regulation, transcriptional plasticity, and non-histone protein regulation, we conceptualize the HDAC–lactylation axis as a dynamic metabolic–epigenetic interface that shapes context-dependent cancer therapy adaptation (Figure 1) [7,12,21]. We distinguish substrate-specific functional evidence from broader associations with tumor progression and identify the mechanistic questions that remain unresolved. The following section outlines lactate metabolism and transport as the metabolic context for understanding these regulatory mechanisms.

## 2. Metabolic Basis of Lactate Accumulation in Resistant Tumors

### 2.1. Enhanced Glycolysis and Lactate Overproduction in Resistant Tumors

Many chemotherapy-resistant cancer models exhibit enhanced aerobic glycolysis, characterized by increased glucose utilization and lactate production even when oxygen is available [22,23,24]. Rather than reflecting impaired oxidative metabolism, this shift provides cancer cells with rapid ATP and metabolic intermediates that support biosynthesis and stress adaptation during treatment [5,22,23]. This phenomenon, commonly referred to as the Warburg effect, differs from hypoxia-induced glycolysis, in which reduced oxygen availability promotes greater reliance on glycolytic metabolism [5]. Although these processes arise under different oxygen conditions, they may coexist within the same tumor and both contribute to lactate production [25,26]. Lactate dehydrogenase A (LDHA) converts pyruvate to lactate while regenerating NAD^+^, thereby supporting glycolytic flux under both oxygen-available and hypoxic conditions [8,23,24,27,28]. Under hypoxic conditions, HIF-1α further promotes glycolytic adaptation by inducing genes involved in glucose uptake and glycolysis, including GLUT1, HK2, and LDHA [8,27,28,29].

Chemotherapy-associated metabolic stress can influence glycolysis and lactate production through both acute adaptive responses and the selection of resistant cells with altered metabolic phenotypes. Among DNA-damaging agents, cisplatin has been reported to enhance glycolytic activity and lactate secretion in HeLa cervical cancer cells through acetylation-dependent cytoplasmic accumulation of PFKFB3 and its subsequent activation by AMPK [30]. In ovarian cancer, cisplatin-resistant patient-derived organoids exhibit enhanced glycolytic metabolism, including increased expression of HK2, GLUT1, and LDHA, although these observations do not establish that acute cisplatin exposure directly induces these changes [31]. Etoposide offers a further example, with ROS-dependent HIF-1α signaling reported to promote glycolysis and lactate production in non-small-cell lung cancer cells [32]. Among antimetabolites, 5-fluorouracil-resistant colorectal cancer cells show enhanced glycolysis and pentose phosphate pathway activity associated with HIF-1α upregulation through ROS/PI3K/Akt and Wnt/β-catenin signaling [33]. For mitotic poisons, paclitaxel treatment and acquired resistance have been associated with increased lactate production and elevated LDHA and MCT1 expression in breast cancer models [34]. These findings point to several mechanisms, including AMPK–PFKFB3 activation and ROS-associated HIF-1α signaling, through which chemotherapy exposure or resistance may be linked to enhanced glycolytic metabolism. However, treatment-induced metabolic adaptation should be distinguished from the selection of resistant cells, and increased lactate production cannot be assumed to occur uniformly across chemotherapeutic classes. These metabolic changes can sustain lactate production during treatment, creating a metabolic context in which lactate transport, signaling, and protein lactylation may become relevant to chemotherapy response [22,23,35].

### 2.2. Lactate Transport and Metabolic Organization in the Tumor Microenvironment

Highly glycolytic cancer cells rely on lactate-proton transport to limit intracellular acidification and maintain metabolic homeostasis [25]. Although undissociated lactic acid can diffuse across lipid membranes, its contribution to lactate exchange is limited at physiological and typical tumor-associated pH, where lactate predominantly exists in its negatively charged form. Consequently, proton-coupled transport by monocarboxylate transporters (MCTs) provides a major pathway for lactate exchange in tumor cells [36]. Monocarboxylate transporter 4 (MCT4; SLC16A3) is frequently induced under hypoxic conditions and acts as a major route for lactate efflux from highly glycolytic tumor cells [25,37]. Monocarboxylate transporter 1 (MCT1; SLC16A1) can mediate lactate uptake in better-oxygenated tumor cells, allowing lactate to be used as an oxidative fuel, although transport direction depends on lactate and proton gradients [25,26]. This MCT1–MCT4 division of labor supports a form of metabolic symbiosis in which hypoxic cells export lactate and neighboring oxidative cells reutilize it as a carbon source, preserving regional metabolic flexibility under fluctuating oxygen and nutrient availability [25,26,38]. Together, MCT-mediated lactate exchange contributes to metabolic flexibility within tumors and provides a relevant context for understanding how lactate-associated signaling may influence cellular responses to chemotherapy [22,25,39].

### 2.3. Lactate Signaling in Stress Adaptation and Therapy Tolerance

Beyond its role as a metabolic end product, lactate also acts as a signaling metabolite that connects extracellular metabolic conditions to intracellular transcriptional and phenotypic responses [6,39,40]. One well-characterized signaling route involves GPR81 (HCAR1), which has been linked to cancer-cell survival, glycolytic adaptation, and the production of pro-angiogenic factors [6,40,41,42,43]. In experimental cancer models, GPR81 signaling has been shown to promote the expression of pro-angiogenic factors and to support metabolic programs required for tumor-cell growth [6,42,43]. Lactate also intersects with hypoxia-responsive signaling by stabilizing HIF-1α or amplifying HIF-dependent transcription under partially oxygenated conditions, thereby creating a feed-forward loop in which glycolysis drives lactate accumulation, lactate reinforces hypoxia-associated signaling, and HIF-1α further promotes glycolytic and lactate-export programs [37,39,44]. In chemotherapy-treated cells, lactate exposure has been linked to changes in DNA repair capacity and anticancer drug responses in cervical cancer models, with reported involvement of HCAR1 signaling and HDAC inhibition [22,45,46]. These findings concern lactate-associated signaling and HDAC activity; they do not by themselves establish a role for protein lactylation in chemotherapy resistance.

Lactate has also been proposed to exhibit oncometabolic functions in certain cancer contexts, including effects on cancer-associated transcriptional programs and tumor progression [47]. Unlike classical oncometabolites such as D-2-hydroxyglutarate and succinate, lactate is a normal physiological metabolic intermediate that frequently accumulates in tumors through metabolic reprogramming. Its potential classification as an oncometabolite should therefore reflect context-dependent tumor-promoting activity rather than mechanistic equivalence with these classical oncometabolites.

### 2.4. From Lactate Accumulation to Epigenetic Plasticity

Lactate accumulation also has direct consequences for epigenetic regulation, extending its significance beyond metabolism and signaling. In highly glycolytic tumors exposed to therapeutic stress, excess lactate can alter histone modification patterns, chromatin accessibility, and metabolite-responsive transcriptional programs [7,8,48]. The discovery of histone lysine lactylation provides a mechanistic basis for understanding how lactate abundance may be translated into transcriptional regulation, linking metabolic reprogramming to epigenetic adaptation [7,49]. This connection is particularly relevant in resistant tumors, where reversible chromatin remodeling and adaptive transcriptional plasticity allow cancer cells to survive cytotoxic stress without necessarily acquiring fixed genetic changes [3,4,50]. Accordingly, lactate accumulation should be viewed not only as a hallmark of tumor metabolic rewiring but also as a potential upstream regulator of chromatin-associated adaptation [7,48]. This metabolic–epigenetic interface provides the conceptual basis for examining how lactylation and HDAC-dependent regulatory mechanisms may converge to shape adaptive phenotypes associated with chemoresistance in cancer [7,12,22,48]. However, lactate accumulation alone does not establish that specific lactylation events mediate chemotherapy resistance. Evaluating this relationship requires consideration of histone and non-histone substrates and the enzymes regulating lactylation turnover, which are examined in the following section.

## 3. Lactylation as a Metabolism-Driven Regulatory Layer in Cancer

### 3.1. Metabolic Basis of Lactylation in Cancer

The discovery of lysine lactylation established a direct biochemical link between cellular metabolism and protein regulation [7,51]. Lactylation arises from intracellular lactate, with greater lactate availability and glycolytic activity associated with increased lysine lactylation [7,15]. This modification can occur on both histone and non-histone proteins, reflecting the broader capacity of metabolic intermediates to directly influence cellular regulatory systems [17,20]. In cancer, elevated lactate levels are driven by aerobic glycolysis and microenvironmental constraints such as hypoxia, which collectively increase substrate availability for lactylation [7,10,52]. However, lactylation is not determined by lactate availability alone and can arise through enzymatic and non-enzymatic pathways. ACSS2 can generate lactyl-CoA for KAT2A-mediated histone lactylation [14], whereas AARS1 can use a lactyl-AMP intermediate without requiring lactyl-CoA [15]. Lactate exists as two enantiomers, L-lactate and D-lactate, which differ in configuration at the chiral C2 carbon. L-lactate is primarily produced from pyruvate by lactate dehydrogenase during mammalian glycolysis [7], whereas D-lactate is generated at lower levels, notably through methylglyoxal metabolism via the glyoxalase pathway [53]. This pathway produces S-D-lactoylglutathione, which can mediate non-enzymatic lysine D-lactylation, also termed lysine lactoylation [53]. Isomer-resolved analyses indicate that L-lactyllysine is the predominant histone lactylation isomer associated with glycolysis in the systems examined. This distinction highlights the importance of isomer-specific analytical methods and validated antibodies when interpreting lactylation-related mechanisms [54]. Lactyl-CoA is less abundant than major physiological acyl-CoA species in the mammalian systems examined, highlighting the importance of identifying the relevant lactyl-donor pathway [54]. Importantly, lactylation is neither uniform nor constitutive. Its abundance varies with metabolic flux, cellular context, and the activity of regulatory enzymes, indicating that it functions as a responsive rather than static layer of regulation [12,16]. This dynamic nature provides a mechanistic basis for linking metabolic adaptation to downstream regulatory processes in cancer cells [7,12,16,55].

Lactylation also occurs in non-malignant physiological contexts. During macrophage activation, glycolysis-associated histone lactylation contributes to homeostatic gene expression [7]. Histone lactylation has also been implicated in neural crest cell specification and migration during embryonic development [56]. These findings indicate that lactylation is not specific to cancer; its relevance depends on the metabolic and cellular context and the consequences of the modified proteins. This distinction also warrants consideration when evaluating therapeutic interventions targeting lactate metabolism or HDAC-regulated lactylation. The following section examines how histone lactylation influences transcriptional regulation and how it compares with acetylation and other acyl marks.

### 3.2. Histone Lactylation and Transcriptional Adaptation

Histone lactylation has been most extensively characterized as a chromatin-associated modification linked to transcriptional activation. Genome-wide analyses have demonstrated that histone lactylation is enriched at promoter and enhancer regions of actively transcribed genes, suggesting a role in facilitating gene expression [7,57]. Comparison with acetylation and other histone acylations helps clarify the distinctive chemical properties, metabolic inputs, and regulatory consequences of lactylation.

Although histone lysine acetylation (Kac) and lactylation (Kla) both neutralize the positive charge of the lysine ε-amino group, the additional hydroxyl group of the lactyl moiety makes it more hydrophilic and increases its hydrogen-bonding potential. This chemical distinction can influence histone-mark recognition, as illustrated by the different molecular interactions involved in DPF2 recognition of H3K14la and H3K14ac [58]. The hydroxyl group retained on protein-bound lactyl moieties could, in principle, undergo additional enzymatic or chemical modifications, such as O-acylation or phosphorylation. However, currently characterized lactylation writers and delactylases primarily catalyze the installation or removal of the lysine-bound lactyl group rather than secondary modification of its hydroxyl group [7,12,14,15]. To our knowledge, enzyme-catalyzed modification of this hydroxyl group has not been experimentally established, and its potential biological significance remains unknown. Histone acetylation primarily depends on acetyl-CoA, whereas enzymatic lactylation can involve lactyl-CoA or alternative activated lactyl intermediates, as discussed in Section 3.1. Both modifications can involve shared regulatory enzymes, including p300 and HDAC1–3 [7,12]. Nevertheless, their transcriptional consequences may differ in timing, genomic distribution, and molecular recognition. During macrophage activation, histone lactylation accumulates later than acetylation and is associated with the induction of homeostatic genes, including Arg1 [7]. H3K18la also marks subsets of tissue-specific active enhancers [57]. These observations suggest that lactylation and acetylation may participate in distinct transcriptional programs depending on cellular context, although the extent of their independent regulatory functions remains unresolved. Acetyl-CoA availability and histone acetylation have also been reported to change in certain chemotherapy-related models, including cisplatin-resistant cells [59] and chemotherapy-associated NAT10–ACLY signaling in hepatocellular carcinoma [60]. These findings support parallel profiling of acetylation and lactylation when investigating therapy-associated chromatin changes, as further discussed in Section 7.1.

Potential crosstalk between lactate and short-chain fatty acid (SCFA) metabolism can arise at three levels: the availability of activated acyl donors, the use of shared acyltransferases or deacylases, and modification of the same lysine residue. SCFAs, including acetate, propionate, and butyrate, can contribute to distinct cellular acyl-CoA pools [61]. A given lysine residue can carry only one acyl modification at a time, while certain acyltransferases can utilize multiple acyl-CoA donors. Biochemical studies indicate that histone acyltransferases, including p300, discriminate among alternative acyl-CoA donors [62]. Structural and biochemical analyses have also demonstrated acyl-group discrimination by GCN5, including interactions involving butyryl-CoA [63]. However, these findings do not directly establish competition between lactyl-CoA and SCFA-derived acyl-CoAs in cellular models. Additional crosstalk may occur through HDAC regulation: lactate and butyrate can inhibit HDAC deacetylase activity with markedly different potencies, although this does not establish direct competition in delactylation reactions, as discussed in Section 3.4.

Beyond competition for lysine modification sites and sequential deacylation–reacylation, direct enzyme-catalyzed exchange between lysine-bound lactyl and acetyl groups could also be considered theoretically. Established mechanisms involve HDAC-mediated hydrolytic deacylation, NAD^+^-dependent sirtuin-catalyzed deacylation, and enzymatic lactyl-group transfer using activated donors such as lactyl-CoA (p300) or lactyl-AMP (AARS1), each operating through distinct removal or installation steps [7,12,14,15]. Because lysine acyl modifications involve relatively stable amide bonds, direct exchange would require an additional activation step or a suitable catalytic intermediate, potentially involving an acyl–enzyme species. To our knowledge, however, direct enzymatic lactyl–acetyl exchange has not been experimentally established. Whether such a pathway exists remains an open biochemical question.

Other histone acylations further illustrate how differences in acyl-group chemistry influence chromatin regulation. Crotonylation uses crotonyl-CoA and introduces an unsaturated acyl group through p300-mediated modification [64], while crotonylated lysine can be recognized by the AF9 YEATS domain [65]. In contrast, succinylation introduces a negatively charged group, with H3K122 succinylation reported to destabilize nucleosomes and enhance transcription [66]. These examples illustrate differences in metabolic donors, molecular recognition, and chromatin effects without establishing that any single histone acylation universally determines transcriptional outcomes.

Several studies provide locus-specific examples of this transcriptional regulation. For example, H3K18 lactylation has been shown to activate PPARD transcription in breast cancer, linking metabolic state to gene expression programs [67]. Similarly, lactylation at the RUBCNL promoter has been associated with increased transcription of an autophagy-related regulator, highlighting the capacity of lactylation to couple metabolic inputs to gene expression outputs and contribute to bevacizumab resistance, a treatment context distinct from cytotoxic chemotherapy [68]. Additional studies have linked histone lactylation to MYC-associated transcriptional programs and stemness-related gene expression, suggesting a broader role in adaptive cell-state regulation [9,69]. More directly relevant to cytotoxic chemotherapy, H4K12la-associated GCLC expression suppresses ferroptosis and promotes oxaliplatin resistance in colorectal cancer stem cells [70], whereas H3K9la-associated RAD51 and BRCA2 transcription has been linked to platinum resistance in ovarian cancer [71]. Separately, H4K12la-associated RAD23A expression has been implicated in niraparib resistance in ovarian cancer, representing a distinct PARP inhibitor treatment context [72]. Despite these findings, many studies still report associations between lactylation enrichment and altered gene expression, while the causal contribution of individual lactylation marks at specific genomic loci remains incompletely resolved [16,17,20]. Representative examples of histone lactylation-dependent transcriptional regulation in cancer are summarized in Table 1. Together, these findings indicate that histone lactylation participates in context-dependent transcriptional regulation, while site-specific causality remains incompletely established. The following section examines how lactylation extends beyond chromatin to regulate non-histone proteins.

### 3.3. Non-Histone Lactylation: Expanding Functional Regulation

Lactylation has also been identified on a wide range of non-histone proteins, extending its functional scope beyond chromatin regulation. Proteomic analyses have revealed lactylation on proteins involved in metabolism, RNA processing, and signaling pathways, suggesting that this modification may directly influence protein function, stability, and interactions [17,18,51]. Non-histone lactylation has been proposed to modulate enzyme activity, subcellular localization, and protein–protein interactions, although these mechanisms remain less well characterized than histone-associated effects [16,19]. A more defined example is HDAC2-mediated delactylation of METTL3, which has been linked to altered RNA methylation, DNA damage repair, and cisplatin resistance in triple-negative breast cancer [74]. Other HDAC2-associated non-histone substrates, including DHX15 and NUDT21, illustrate the potential influence of delactylation on RNA processing, although their reported functions should not be interpreted as direct evidence of chemotherapy resistance [75,76]. These substrate-specific mechanisms are examined further in Section 5.4.

At present, most studies describing non-histone lactylation focus on mapping modification sites and associated pathways, with relatively limited mechanistic resolution at the level of individual substrates. Thus, although non-histone lactylation is increasingly recognized as a widespread phenomenon, its functional consequences in specific cellular contexts remain incompletely defined [17,18,19]. Understanding how these modifications are enzymatically regulated provides an important basis for evaluating their functional significance, as discussed in the following section.

### 3.4. Enzymatic Regulation of Lactylation Dynamics

Enzymatic control of lactylation involves proteins that add or remove lactyl groups, commonly referred to as “writers” and “erasers” [7,12,17]. The histone acetyltransferase p300 has been shown to catalyze histone lactylation, indicating that enzymes traditionally associated with acetylation can also mediate lactylation under appropriate metabolic conditions [7,13]. Conversely, class I histone deacetylases (HDAC1–3) have been identified as histone lysine delactylases, demonstrating that lactylation is subject to active enzymatic removal [12]. This finding establishes lactylation as a reversible modification and introduces a dynamic regulatory axis in which deposition and removal are regulated by multiple enzymes. Although HDAC1–3 exhibit delactylase activity in biochemical assays, their activities toward individual histone sites and cellular substrates can differ [12]. NAD^+^-dependent sirtuins, including SIRT1–3 and SIRT6, also exhibit delactylase activity [77,78]. The present review focuses primarily on HDAC1–3 to examine their substrate-specific regulatory functions and evaluate whether HDAC-associated changes in lactylation contribute to chemotherapy resistance or sensitization, while acknowledging the broader roles of sirtuins. Biochemical evidence further indicates that HDAC1–3 can catalyze lysine lactylation under specific experimental conditions [79], indicating that their catalytic functions can extend beyond lactyl-group removal.

Lactate concentrations vary with tissue type, oxygen availability, and the compartment measured, although quantitative data across these conditions remain limited. Physiological lactate levels in blood and healthy tissues are often reported at approximately 1.5–3 mM, whereas values of 10–30 mM have been described in certain tumor tissues [80]. However, reliable compartment-specific ranges for healthy tissues are less well established, and whole-tissue measurements cannot be equated with intracellular or extracellular concentrations. For example, quantitative bioluminescence imaging of human breast tissue specimens revealed median lactate values ranging from 0.6 to 8.0 µmol/g in locally advanced breast cancer biopsies, compared with 0.8 to 1.9 µmol/g in benign breast tissue, highlighting substantial heterogeneity in tissue lactate accumulation [81]. Intracellular measurements also vary considerably: genetically encoded biosensors indicated baseline lactate levels in the hundreds of micromolar range in cultured HeLa cells, increasing to at least 1 mM following MCT1/2 inhibition, although these estimates do not represent concentrations in intact tumors [82]. Oxygen availability further influences lactate accumulation; in ME180 tumor xenografts, exposure of tumor-bearing mice to 7% oxygen for 3 h modestly increased whole-tissue lactate content from 12.1 ± 0.86 to 14.9 ± 0.89 µmol/g (*p* = 0.05) [83]. Within heterogeneous tumors, relatively oxygenated cells may also import and oxidize lactate released by glycolytic cells through monocarboxylate transporters, contributing to metabolic symbiosis [84]. Nevertheless, universal intracellular and extracellular lactate ranges for normoxic and hypoxic tumor regions remain insufficiently established.

Lactate has also been reported to inhibit HDAC activity through a mechanism distinct from protein lactylation. In a cell-free assay using a HeLa nuclear extract and an acetylated substrate, L-lactate inhibited HDAC activity with an IC_50_ of approximately 40 mM, compared with approximately 50 μM for butyrate [85]. In cervical carcinoma models, L- and D-lactate exposure has been associated with reduced HDAC activity, increased histone acetylation, and changes in DNA repair and anticancer drug responses [45,46]. These findings concern inhibition of HDAC deacetylase activity and do not demonstrate direct inhibition of HDAC-mediated delactylation by lactate.

The effects of butyrate on histone delactylation require separate consideration. Sodium butyrate treatment increased global histone lactylation in HeLa cells, but its direct effect on HDAC delactylase activity was not tested biochemically; delactylase inhibition was assessed using other HDAC inhibitors [12]. In contrast, butyrate treatment reduced global lysine lactylation in HEK293T cells in a study demonstrating that HDAC1–3 can also catalyze lactylation [79]. These contrasting cellular observations indicate that changes in lactylation following butyrate treatment cannot be attributed solely to inhibition of delactylation. Direct inhibition of HDAC-mediated delactylation by butyrate has not been demonstrated in these studies.

The balance between lactylation and delactylation is likely influenced by both metabolic inputs and enzyme activity, suggesting that lactylation operates within a broader regulatory network that integrates metabolism with chromatin and protein regulation [7,12,17,86]. In this context, HDACs function as key components of enzymatic machinery controlling lactylation turnover, rather than as direct determinants of specific phenotypic outcomes [12,17]. The functional consequences of lactylation depend on substrate-specific deposition and removal, and changes in HDAC activity should not be interpreted as evidence of lactylation-mediated phenotypes without further validation. This framework provides a basis for examining how HDAC-mediated regulation of lactylation may influence adaptive cellular states in cancer, as discussed in the following sections.

### 3.5. Lactylation-Mediated Regulation of Immune Cells in the Tumor Microenvironment

The immunological consequences of histone lactylation are not uniform across the tumor microenvironment (TME), but depend on the immune-cell population and transcriptional programs involved. In ovarian cancer, lactate exposure increased H3K18 lactylation at the CCL18 promoter in macrophages, accompanied by elevated CCL18 expression and enhanced proliferation and migration of cocultured cancer cells [87]. Similarly, in glioblastoma, glucose-driven lactate production promoted histone lactylation and IL-10 expression in monocyte-derived macrophages, contributing to the suppression of T-cell activity [88]. These findings implicate macrophage lactylation in tumor-supportive immune programs. In contrast, histone lactylation in CD8^+^ T cells has been associated with regulatory programs supporting T-cell activation and effector function, with experimental modulation of H3K18la and H3K9la influencing antitumor immunity in preclinical models [89]. Collectively, these observations indicate that lactylation-associated immune regulation can favor either immunosuppressive or antitumor responses, depending on cellular context.

Importantly, these lactylation-associated effects on tumor-supportive immunity and antitumor responses can occur independently of chemotherapy. They therefore provide evidence of broader tumor fitness and immune regulation, but do not by themselves establish a mechanism of chemoresistance. Nevertheless, chemotherapy exposure can alter tumor-cell glycolysis and lactate production in some experimental models [24], potentially modifying lactylation-dependent responses under treatment pressure. Because HDACs regulate lactylation turnover and represent pharmacologically targetable enzymes, an important question is whether HDAC-regulated lactylation makes an additional contribution to chemotherapy adaptation beyond its effects in untreated tumors. Comparing untreated and chemotherapy-exposed models, together with substrate-specific lactylation and functional response measurements, could help distinguish baseline tumor-supportive functions from treatment-associated resistance mechanisms. The following section examines how HDAC-dependent chromatin and protein regulation may contribute to chemotherapy adaptation.

## 4. HDAC-Mediated Epigenetic Regulation in Cancer

### 4.1. Chromatin Remodeling and Chromatin State Reversibility

Histone deacetylases (HDACs) regulate chromatin architecture by removing acetyl groups from lysine residues on histone tails, an activity that generally reduces local chromatin accessibility and alters transcriptional competence [90]. This activity is not simply repressive in a static sense. In cancer cells, HDAC-dependent deacetylation helps maintain chromatin in a dynamic and reversible state, allowing promoter and enhancer regions to be reconfigured in response to therapeutic and environmental stress [50,90,91].

Mechanistically, class I HDACs are closely linked to nuclear co-repressor complexes that shape promoter accessibility and transcriptional output, whereas other family members contribute to broader chromatin regulation in specific cellular compartments [90,92]. This reversibility is particularly relevant in cancer, where non-genetic adaptation often depends on chromatin states that can be re-entered or exited without stable mutation [50]. Studies in lung cancer and glioblastoma have shown that reversible epigenetic states can support drug tolerance and may remain vulnerable to epigenetic intervention [50,93]. HDAC activity has also been implicated in the regulation of therapeutic resistance in both tumor types [94,95].

Accordingly, HDAC-mediated chromatin remodeling is best viewed as a framework for adaptive regulation rather than a simple off-switch for transcription. By modulating how accessible or restrictive chromatin remains under stress, HDACs help define the range of transcriptional states available to cancer cells during therapy adaptation [90,91,96]. These changes in chromatin accessibility provide a basis for understanding how HDACs contribute to transcriptional plasticity, as discussed in the following section.

### 4.2. Transcriptional Regulation and Cell State Plasticity

The reversible chromatin states maintained by HDACs translate into transcriptional plasticity, enabling cancer cells to shift between phenotypic programs under therapeutic pressure. This is especially evident in drug-tolerant persister states, lineage switching, and other non-genetic adaptive transitions in which gene expression changes precede or substitute for stable genetic resistance mechanisms [2,3,68,97]. HDACs participate in this process not only by controlling histone acetylation at regulatory elements but also by functioning within co-repressor complexes and by modulating the activity of transcription-associated proteins, thereby enabling rapid reorganization of gene expression programs [92,97,98]. In therapy-adapted cancer cells, these changes often involve the transient activation of survival, stemness, or dedifferentiation programs rather than permanent lineage commitment [2,3,99,100]. Studies across multiple tumor types now support the view that HDAC activity contributes to these reversible state transitions and helps maintain a pool of cells capable of surviving otherwise lethal treatment [50,98]. For chemoresistance, the key point is that HDACs do more than suppress individual genes. By shaping the flexibility of transcriptional networks, they can help cancer cells enter and maintain adaptive states that remain reversible but are nevertheless clinically important [1,3,50,98].

### 4.3. Non-Histone Protein Regulation as an Extension of Epigenetic Control

Although HDACs were first defined through their actions on histones, their regulatory scope extends well beyond chromatin. A large body of work has shown that HDACs also deacetylate non-histone substrates, including transcription factors, DNA repair proteins, chaperones, and signaling regulators, thereby modifying protein stability, localization, interaction networks, and functional output [101,102]. Thus, HDAC-mediated regulation in cancer is not limited to chromatin compaction but also operates through the direct control of proteins involved in transcription and stress responses [97,101].

Representative examples illustrate this broader logic. HDAC6-mediated deacetylation of α-tubulin influences cytoskeletal organization and cell motility, while deacetylation of HSP90 alters chaperone function and can affect oncogenic signaling networks [103,104]. Studies of p53, STAT3, and NF-κB p65 further show that HDAC-dependent deacetylation can alter transcription-factor stability, dimerization, DNA binding, and transcriptional activity without requiring an initial change in histone marks [87,105,106].

In the context of therapy adaptation, this non-histone layer matters because it links HDAC activity to DNA repair capacity, stress signaling, and survival pathways that often cooperate with chromatin-based plasticity [104,107,108]. Thus, HDACs should be understood as multilayer regulators whose functions extend from chromatin regulation to the direct control of non-histone proteins that shape adaptive cell behavior [16,109,110,111].

### 4.4. Crosstalk Between HDACs and Non-Coding RNAs

A further layer of HDAC-mediated regulation involves crosstalk with non-coding RNAs, particularly microRNAs and long non-coding RNAs. This relationship is bidirectional: non-coding RNAs can regulate HDAC expression or recruitment, while HDAC-dependent chromatin states can in turn influence non-coding RNA transcription [112,113,114]. In cancer, such crosstalk can reinforce transcriptional programs associated with stemness, invasion, and treatment adaptation [112,113,115]. This layer should not be overinterpreted as a separate mechanism independent of chromatin regulation. Rather, it is better understood as an amplifying and stabilizing network superimposed on HDAC-controlled transcriptional states. Studies in pancreatic cancer, breast cancer, and oral or head-and-neck squamous cell carcinoma have linked ncRNA circuits to stemness, chemoresistance, and treatment response. In breast and squamous cell carcinoma models, these circuits have also been shown to intersect directly with HDAC-dependent regulation [114,116,117,118]. Finally, these findings support a broad but coherent view of HDAC-mediated epigenetic regulation in cancer: HDACs shape chromatin accessibility, preserve transcriptional plasticity, regulate functionally important non-histone proteins, and participate in non-coding RNA regulatory circuits. This framework provides the conceptual basis for the next section, in which lactylation is considered as an additional regulatory layer that intersects with HDAC-dependent plasticity in chemoresistant cells.

## 5. The HDAC–Lactylation Axis in Chemoresistance: A Metabolic–Epigenetic Interface

### 5.1. HDAC-Mediated Regulation of Lactylation Dynamics in Cancer Cells

Lactate accumulation is a hallmark of tumor metabolic reprogramming and is directly linked to the formation of lysine lactylation, a metabolite-derived post-translational modification [7,119]. Lactylation is a dynamically regulated and reversible modification, with its abundance influenced by both lactate availability and enzymatic turnover [7,10,11,12]. As discussed in Section 3.4, HDAC1–3 exhibit delactylase activity and can also catalyze lysine lactylation under specific biochemical conditions, indicating that their effects depend on substrate availability and reaction context [12,79].

In cancer cells, where elevated glycolytic flux sustains high intracellular lactate levels, the HDAC–lactylation axis represents a metabolic–epigenetic interface linking cellular metabolism to gene regulation [7,12,119]. Histone lactylation-associated gene regulation also occurs in the absence of chemotherapy [7], while HDAC-dependent epigenetic plasticity has been implicated in cancer-cell adaptation under therapeutic stress [50,98]. In evaluating chemotherapy resistance, a key question is whether specific HDAC-regulated lactylation events are functionally linked to altered drug sensitivity, rather than being inferred solely from changes in lactylation abundance or broader cancer-associated phenotypes. Together, biochemical studies establish that HDAC1–3 can regulate lactylation turnover [12,79], while the contribution of specific HDAC-regulated lactylation events to chemotherapy response requires evaluation in defined molecular and treatment contexts. Section 5.2 examines histone lactylation-associated transcriptional programs and the evidence connecting them to treatment response.

### 5.2. HDAC–Lactylation Control of Transcriptional Plasticity

At the chromatin level, histone lactylation is enriched at transcriptionally active promoters and enhancers and is generally associated with increased gene expression. In this context, HDAC-mediated delactylation provides a mechanism for constraining or reversing lactylation-dependent transcriptional activation, thereby adding temporal control to metabolically responsive gene expression programs [7,12]. This balance is particularly relevant in cancer cells exposed to metabolic stress, where fluctuations in lactate availability can influence chromatin states and transcriptional output [7,67]. Treatment-associated changes in these processes are considered below in the context of chemotherapy response [70,120]. By regulating lactylation turnover, HDACs may influence the persistence of lactylation-associated transcriptional responses, depending on the chromatin locus and cellular context [12].

Evidence from multiple cancer models suggests that histone lactylation contributes to tumor-associated transcriptional programs beyond chemotherapy response. For example, H3K18 lactylation (H3K18la) has been linked to activation of the PPARD axis under anaerobic metabolic conditions [67] and to immune escape in lung cancer [9], whereas H3K27 lactylation has been associated with stemness-related transcriptional programs in gastric cancer [121]. These findings illustrate functions of histone lactylation that are not necessarily dependent on chemotherapy exposure. More direct treatment-related evidence comes from colorectal cancer stem cells, where p300-mediated H4K12 lactylation (H4K12la) enhances GCLC expression, suppresses ferroptosis, and promotes oxaliplatin resistance, whereas HDAC1-mediated delactylation counteracts this modification [70]. In contrast, in cisplatin-resistant bladder cancer, class I HDAC inhibition with entinostat increases H3K18la-associated DHRS2 expression and restores cisplatin sensitivity [120]. Although this study implicates H3K18la in the sensitization mechanism, entinostat also inhibits deacetylase activity, and contributions from concurrent changes in acetylation or other HDAC-dependent processes cannot be excluded on the basis of HDAC inhibitor treatment alone.

Overall, these findings distinguish the broader roles of histone lactylation in transcriptional regulation from its treatment-specific functions. Importantly, the two chemotherapy models imply opposite consequences for HDAC targeting: HDAC1-mediated removal of H4K12la counteracts a resistance-associated pathway in colorectal cancer stem cells [70], whereas class I HDAC inhibition enhances cisplatin sensitivity in bladder cancer [120]. Although HDAC1–3 delactylase activity is biochemically established [12,79], the consequences of targeting HDACs for chemotherapy response may vary with the modified site, target gene, tumor type, and treatment context. The relative contributions of delactylation and concurrent changes in acetylation also remain to be resolved. Section 5.3 examines the molecular pathways linking histone lactylation to chemotherapy resistance or sensitization.

### 5.3. Transcription-Dependent Adaptive Programs in Chemoresistance

Histone lactylation has been linked to transcriptional programs involved in chemotherapy resistance, with distinct mechanisms reported across cancer models. One mechanism involves metabolic adaptation and redox homeostasis. In colorectal cancer stem cells, H4K12 lactylation at the GCLC promoter increases GCLC expression, thereby promoting glutathione synthesis and resistance to ferroptosis. As outlined in Section 5.2, p300-mediated H4K12la is counteracted by HDAC1-mediated delactylation. Inhibition of LDHA or p300 reduces H4K12la and enhances oxaliplatin sensitivity, while GCLC inhibition promotes ferroptosis and further sensitizes these cells to oxaliplatin [70].

A complementary mechanism has been reported in hepatocellular carcinoma. In that study, the authors established oxaliplatin- and 5-fluorouracil-resistant HCC-LM3 and Huh7 cell lines through repeated drug exposure and selection [122]. Resistant cells exhibited increased glycolytic activity and histone H3K14 lactylation (H3K14la). H3K14la-associated upregulation of NEDD4 promoted PTEN ubiquitination and degradation, leading to activation of PI3K/Akt/mTOR signaling. Reduced PTEN also reinforced glycolysis, forming a positive feedback loop that supported drug resistance. Restoring PTEN reduced intracellular lactate, H3K14la, and NEDD4 levels, providing experimental support for this feedback mechanism. Because resistant lines were generated by repeated drug selection, however, chemotherapy-induced metabolic reprogramming cannot be distinguished conclusively from the selection of pre-adapted cells. Moreover, this study did not identify an HDAC responsible for regulating H3K14la.

Other studies suggest that the transcriptional consequences of the HDAC–lactylation axis are context-dependent and can either restrain or reinforce adaptive cancer phenotypes [120,123]. In lung cancer, SATB2 recruits HDAC3 to the LCN2 promoter, where HDAC3 catalyzes H3K9 delactylation and represses LCN2 transcription [123]. Through this pathway, SATB2–HDAC3-dependent delactylation limits an LCN2-associated transcriptional program that promotes tumor growth and metastasis. This finding indicates that HDAC-mediated delactylation can suppress oncogenic transcription at specific loci [123]. However, the principal functional outcomes in this study concern tumor progression rather than chemotherapy response.

Conversely, in cisplatin-resistant bladder cancer, pharmacological inhibition of class I HDACs with entinostat promotes H3K18la-dependent DHRS2 expression and enhances DHRS2 nuclear translocation. These effects suppress the AKR1C3–androgen axis and restore cisplatin sensitivity [120]. As outlined in Section 5.2, the effects of entinostat cannot be assigned exclusively to delactylation because HDAC inhibition also affects acetylation and other HDAC-dependent processes.

Together, these findings support functional links between specific histone lactylation-associated transcriptional pathways and chemotherapy response: H4K12la–GCLC contributes to ferroptosis resistance [70], H3K14la–NEDD4–PTEN supports a glycolysis-associated feedback loop in drug-resistant hepatocellular carcinoma [122], and H3K18la–DHRS2 is associated with restored cisplatin sensitivity following class I HDAC inhibition [120]. In contrast, the HDAC3–H3K9la–LCN2 pathway has been characterized primarily in tumor progression models [123]. Whether chemotherapy directly induces the entire sequence of lactate accumulation, HDAC-regulated lactylation, and resistance, remains unresolved. Section 5.4 examines HDAC-regulated non-histone lactylation and its potential effects on chemotherapy response through direct regulation of protein function beyond transcription.

### 5.4. Direct Regulation of Chemoresistance-Associated Effectors Beyond Transcription

In addition to transcription-dependent mechanisms, the HDAC–lactylation axis can directly regulate protein function through non-histone lactylation. These modifications can alter protein activity and molecular interactions, providing mechanisms beyond histone-dependent transcriptional regulation [124]. A representative example is the HDAC2–METTL3 axis in triple-negative breast cancer, where lactylation of METTL3 at K27 impairs its m^6^A methyltransferase function, whereas HDAC2-mediated delactylation restores METTL3 function, enhances repair capacity, and contributes to cisplatin resistance [74]. Specifically, HDAC2-mediated METTL3 delactylation promotes its interaction with WTAP, increases m^6^A modification of DNA repair-associated transcripts, and supports their expression [74]. Notably, intracellular lactate concentrations did not differ significantly between parental and cisplatin-tolerant cells, indicating that resistance in this model was associated with altered enzymatic regulation of METTL3 lactylation without a detectable difference in lactate abundance [74].

Beyond DNA-repair proteins, HDAC-regulated non-histone lactylation can also affect RNA-processing machinery. In bladder cancer, HDAC2-mediated delactylation of the RNA helicase DHX15 at K17 alters alternative splicing and reduces the mature RPL9 transcript, thereby promoting tumor progression [75]. Another example involves AARS1, an alanyl-tRNA synthetase whose canonical function is the ATP-dependent attachment of alanine to tRNAᴬˡᵃ during protein synthesis [125]. Beyond this canonical activity, AARS1 has been reported to function as a noncanonical lactyltransferase by activating lactate through an ATP-dependent lactyl-AMP intermediate, analogous to the amino acid activation step of tRNA aminoacylation, and transferring the lactyl group to protein lysine residues without requiring lactyl-CoA, in contrast to lactyl-CoA-dependent lactylation pathways involving p300 [15,126]. In esophageal squamous cell carcinoma, AARS1-mediated lactylation of NUDT21 at K23 enhances its interaction with CPSF6 and promotes lengthening of the FDX1 3′ UTR, thereby reducing FDX1 protein output and increasing resistance to cuproptosis. HDAC2 acts as the opposing delactylase in this pathway [76]. These findings extend the known substrate range of HDAC-regulated lactylation, although the DHX15 and NUDT21 studies do not directly establish resistance to conventional cytotoxic chemotherapy [75,76].

**Figure 2:**
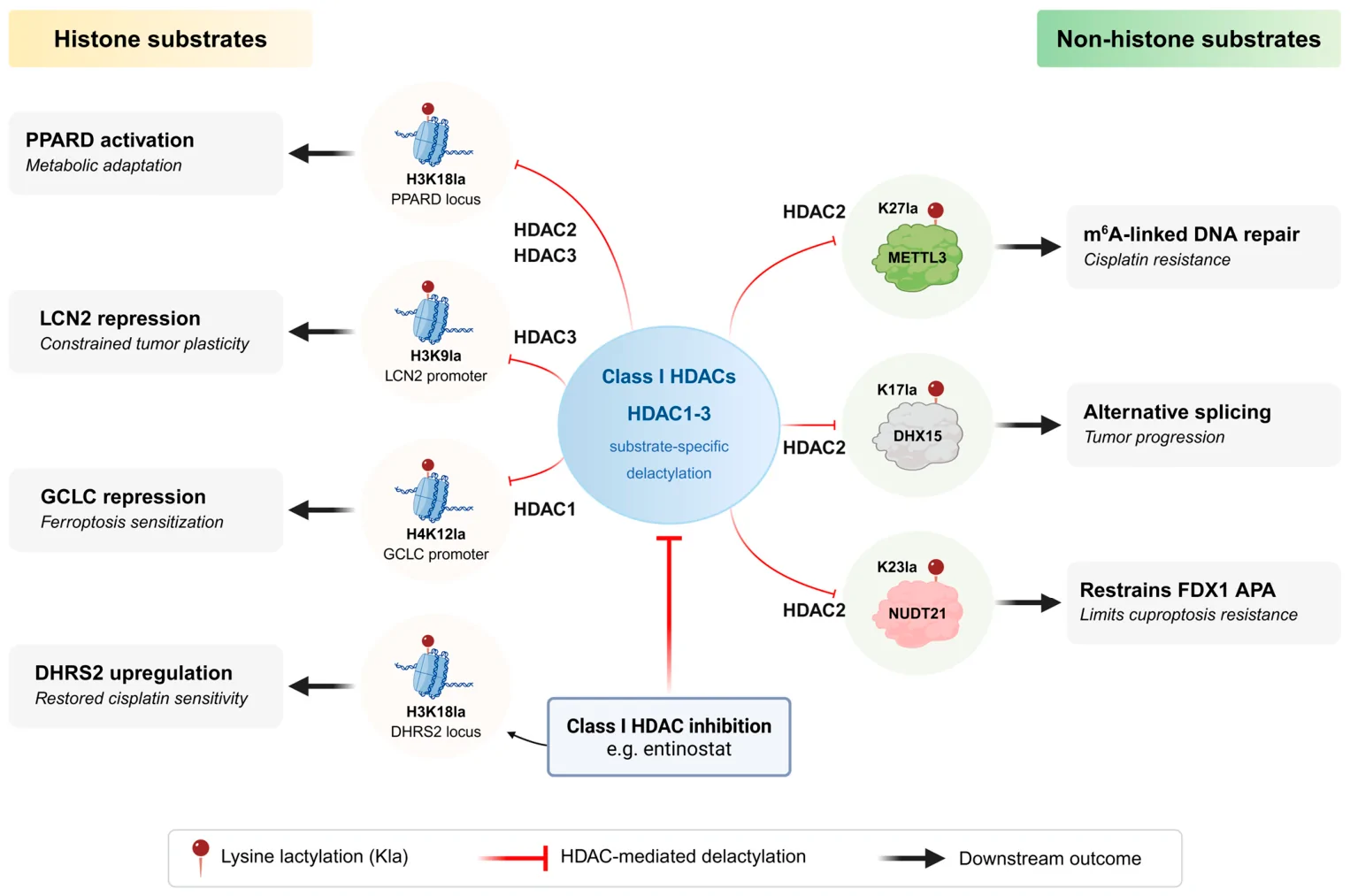
Representative histone and non-histone lactylation mechanisms in cancer biology and treatment response. Class I HDACs regulate lactylation of histone and non-histone substrates, thereby modulating transcriptional programs, DNA repair, RNA processing, and treatment response. The corresponding detailed mechanisms are summarized in Table 2. Abbreviations: HDAC, histone deacetylase; Kla, lysine lactylation; H3K18la, histone H3 lysine 18 lactylation; H3K9la, histone H3 lysine 9 lactylation; H4K12la, histone H4 lysine 12 lactylation; PPARD, peroxisome proliferator-activated receptor delta; LCN2, lipocalin 2; GCLC, glutamate-cysteine ligase catalytic subunit; DHRS2, dehydrogenase/reductase 2; METTL3, methyltransferase 3; DHX15, DEAH-box helicase 15; NUDT21, nudix hydrolase 21; FDX1, ferredoxin 1; m6A, N6-methyladenosine; APA, alternative polyadenylation; DNA, deoxyribonucleic acid; K17la, lysine 17 lactylation; K23la, lysine 23 lactylation; K27la, lysine 27 lactylation. (Created in BioRender. Nguyen, E. (2026) https://biorender.com/dx08mja, accessed on 4 November 2026).

Taken together, these studies illustrate how HDAC-regulated non-histone lactylation can directly affect DNA repair and RNA-processing pathways. Evidence linking this mechanism to cisplatin resistance in this section rests on a single substrate-specific study involving METTL3 [74]. The HDAC2–METTL3 model associates cisplatin resistance with reduced METTL3 lactylation without a significant change in lactate abundance [74], whereas the H4K12la–GCLC model described in Section 5.3 links increased histone lactylation to oxaliplatin resistance [70]. These contrasting findings indicate that the direction of the lactylation–resistance relationship differs between the investigated models, which involve distinct substrates, tumor types, and chemotherapeutic agents; the determinants of these differences remain to be established. Overall, non-histone lactylation represents an additional regulatory layer that complements histone-associated transcriptional mechanisms [75,76,124], with demonstrated effects on chemotherapy response in selected models (Figure 2) [74]. Section 6 examines the therapeutic implications of targeting lactate metabolism and HDAC activity in light of these substrate-specific mechanisms.

**Table 2:** Representative histone and non-histone mechanisms of the HDAC–lactylation axis in context-dependent cancer therapy adaptation.

Biological Process	Lactylated Histone/Protein	HDAC Isoform(s)	Functional Consequence
Ferroptosis regulation	H4K12la/GCLC [70]	HDAC1	HDAC1-mediated delactylation reduces H4K12la and GCLC transcription, promotes ferroptosis and increases chemosensitivity
Metabolic adaptation	H3K18la/PPARD [67]	HDAC2 and HDAC3	Reduced HDAC2/HDAC3-mediated delactylation permits H3K18la accumulation and activates PPARD-associated metabolic adaptation
Phenotypic plasticity/metastatic adaptation	H3K9la/LCN2 [123]	HDAC3	HDAC3-mediated delactylation represses LCN2 transcription and constrains tumor growth/metastatic plasticity
Chemosensitivity reprogramming	H3K18la/DHRS2 [120]	Class I HDAC inhibition	Entinostat promotes H3K18la-mediated DHRS2 expression and restores cisplatin sensitivity
DNA damage repair	METTL3/K27la [74]	HDAC2	HDAC2-mediated delactylation restores METTL3 activity and enhances DNA damage repair
RNA processing/post-transcriptional adaptation	DHX15/K17la [75]	HDAC2	HDAC2-mediated delactylation alters alternative splicing and promotes tumor progression; a direct contribution to conventional chemotherapy resistance remains unestablished
Cuproptosis resistance	NUDT21/K23la [76]	HDAC2	HDAC2-mediated delactylation restrains NUDT21-dependent alternative polyadenylation and limits cuproptosis resistance

Abbreviations: HDAC, histone deacetylase; H4K12la, histone H4 lysine 12 lactylation; H3K18la, histone H3 lysine 18 lactylation; H3K9la, histone H3 lysine 9 lactylation; GCLC, glutamate-cysteine ligase catalytic subunit; PPARD, peroxisome proliferator-activated receptor delta; LCN2, lipocalin 2; DHRS2, dehydrogenase/reductase 2; METTL3, methyltransferase 3; DHX15, DEAH-box helicase 15; NUDT21, nudix hydrolase 21; K27la, lysine 27 lactylation; K17la, lysine 17 lactylation; K23la, lysine 23 lactylation; DNA, deoxyribonucleic acid; RNA, ribonucleic acid.

The HDAC–lactylation axis represents a dynamic metabolic–epigenetic interface that integrates chromatin-based transcriptional regulation with direct protein-level control [7,12,74,75,76,124]. Increased lactate availability can promote lactylation, whereas HDACs regulate lactylation turnover on histone and selected non-histone substrates, with consequences that depend on the modified substrate and cellular context [7,12,74,75,76]. Evidence from specific cancer models links these transcriptional and protein-level mechanisms to chemotherapy resistance or restored treatment sensitivity [70,74,120], while other HDAC-regulated lactylation pathways have primarily been investigated in tumor progression or non-chemotherapy-related responses [75,76]. It is therefore better understood as a multilayered regulatory network rather than a linear pathway. This framework provides a rationale for exploring lactylation-directed approaches in combination with existing therapies [119,127]. To further illustrate this concept, representative HDAC-linked histone and non-histone lactylation mechanisms underlying context-dependent cancer therapy adaptation are summarized in Table 2.

## 6. Therapeutic Targeting of the HDAC–Lactylation Axis

The emerging recognition of lactylation as a metabolite-driven regulatory mechanism has prompted increasing interest in its potential therapeutic relevance in cancer. Approaches under investigation include targeting lactate metabolism and HDAC activity, although their effects on lactylation-dependent chemotherapy responses remain supported mainly by mechanistic and preclinical evidence [128,129]. The HDAC–lactylation axis is therefore a framework for understanding how metabolic and epigenetic interventions may intersect; its therapeutic targeting has not yet been clinically validated.

One strategy under investigation is to target lactate metabolism, which provides the upstream substrate for lactylation. LDHA inhibition can reduce lactate production and, in colorectal cancer stem cells, decrease H4K12 lactylation and enhance oxaliplatin sensitivity [70]. Pharmacological inhibition of MCT1 or genetic suppression of MCT4 can disrupt lactate exchange and tumor metabolic homeostasis, although the consequences for intracellular lactate depend on transport direction and cellular context [130]. However, the extent to which therapeutic effects of metabolic inhibition are specifically mediated through changes in lactylation, rather than broader metabolic disruption, remains unclear [128,129]. Determining this contribution requires measurement of intracellular lactate, site-specific lactylation, and chemotherapy response following metabolic intervention. Because lactate and short-chain fatty acids such as butyrate can also inhibit HDAC deacetylase activity (Section 3.4), metabolic interventions should be assessed for effects on HDAC activity independently of their effects on protein lactylation.

A second strategy involves epigenetic regulators, particularly HDACs. The identification of HDAC1–3 as delactylases suggests that pharmacological modulation of these enzymes could alter lactylation dynamics [12]. HDAC inhibitors are clinically approved for selected hematological malignancies, but their effects on lactylation cannot be predicted solely from their established effects on acetylation [131,132]. Because class I HDACs can remove lactyl groups, their inhibition may increase specific lactylation marks [12,70,120]. However, the biological outcome is not uniform: increased lactylation has been associated with both chemoresistance and restored treatment sensitivity, depending on the substrate, genomic locus, and tumor context [70,120]. Furthermore, HDAC2-mediated METTL3 delactylation has been linked to cisplatin resistance, indicating that reduced lactylation can also accompany a resistant phenotype in a defined experimental model [74]. HDAC inhibitors also affect acetylation, and HDAC1–3 can catalyze lysine lactylation under specific biochemical conditions [79].

Accordingly, the therapeutic consequences of HDAC inhibition must be evaluated in a substrate- and context-specific manner [128,131]. Isoform-focused pharmacological and genetic perturbation could help identify which HDAC regulates individual lactylation sites. RGFP966 has been used as an HDAC3-selective inhibitor, but kinetic analyses have demonstrated substantial inhibition of HDAC1 and HDAC2, limiting its suitability as an HDAC3-specific probe [133]. Pharmacological studies should therefore verify isoform selectivity and target engagement and incorporate genetic loss-of-function and rescue experiments. Such approaches remain tools for preclinical mechanistic investigation and should not be interpreted as clinically validated strategies for targeting lactylation.

These considerations highlight the value of investigating combination strategies that simultaneously target metabolic and epigenetic layers. For instance, co-targeting lactate production and chromatin regulators may provide a means to modulate both the generation and turnover of lactylation signals. Although such approaches remain largely hypothetical, they are conceptually supported by the interdependence of metabolic state and chromatin regulation in cancer cells [134]. However, there is still limited direct evidence that combined targeting of lactate metabolism and HDAC activity improves therapeutic outcomes specifically through modulation of lactylation [128,129]. Proposed combinations should be compared with chemotherapy alone and with individual metabolic or HDAC-directed interventions, while monitoring changes in lactylation and acetylation.

Taken together, targeting lactate metabolism and HDAC activity offers complementary approaches to modulating the metabolic and epigenetic processes associated with chemotherapy response. However, the therapeutic consequences of these interventions depend on the relevant lactylation substrates, HDAC isoforms, and tumor contexts, as illustrated by the contrasting findings involving H4K12la–GCLC, H3K18la–DHRS2, and HDAC2–METTL3 [70,74,120]. Combining metabolic and HDAC-directed interventions may provide additional opportunities to influence treatment response, but whether such combinations improve chemotherapy sensitivity through specific changes in lactylation remains to be established. Distinguishing lactylation-dependent effects from broader alterations in metabolic flux, acetylation, and other HDAC-regulated processes will therefore be essential for evaluating their therapeutic relevance. These considerations also highlight the importance of identifying the enzymes and substrates responsible for individual treatment-associated phenotypes. Section 7 addresses these challenges by outlining experimental approaches to establish causality, define enzyme–substrate specificity, characterize the persistence of lactylation-associated states, and assess their functional contribution to chemoresistance.

## 7. Future Directions and Unresolved Questions in the HDAC–Lactylation Field

Despite recent advances in defining lactylation as a metabolism-responsive modification of histone and non-histone proteins, the HDAC–lactylation axis remains poorly understood in the context of chemoresistance [49,135]. Returning to the central question of whether HDAC-regulated lactylation causally influences chemotherapy response, four issues remain: (i) causality [10,136], (ii) enzymatic specificity [12,137], (iii) reversibility after treatment withdrawal [136,137], and (iv) recognition by lactylation readers [49,137,138]. Although several studies link lactylation to treatment-associated phenotypes, direct evidence for HDAC-dependent mechanisms in chemotherapy response remains concentrated in selected preclinical models [70,74,120]. The following sections examine these questions and the experimental approaches needed to address them.

### 7.1. Establishing Causality: Driver or Byproduct of Resistant States?

One of the central unresolved issues in the field is whether lactylation actively drives resistance-associated phenotypes or instead reflects the highly glycolytic metabolic state adopted by therapy-stressed tumor cells. Chemotherapy exposure can alter metabolic activity and lactate production, but increased lactate and lactylation should be established experimentally in each model. In some settings, lactylation may therefore serve primarily as a readout of metabolic stress without necessarily directing adaptation [10,110]. Several findings, however, argue against a purely passive role. Histone lactylation is associated with transcriptional regulation [7] and is controlled by defined writers and erasers [7,12,137]. Specific studies have linked lactylation to resistance to bevacizumab [68], oxaliplatin-associated ferroptosis defense [70], niraparib resistance [72], and platinum resistance [71]. These findings involve distinct therapeutic agents and biological mechanisms. The question of causality is therefore closely connected to lactylation turnover. By contributing to lactylation turnover, HDACs may influence whether these modifications remain transient metabolic readouts or persist long enough to reinforce adaptive states [12,70,74,137]. Future studies will therefore need to move beyond steady-state correlations and directly test whether manipulating lactylation turnover, alongside lactate abundance, alters the emergence, maintenance, or reversibility of therapy-tolerant phenotypes [1,3,12,137].

As discussed in Section 5.3, the H3K14la–NEDD4–PTEN pathway links enhanced glycolysis and lactylation to oxaliplatin and 5-fluorouracil resistance, but whether chemotherapy induces this pathway or selects cells with pre-existing pathway activity remains unresolved [122]. Drug-tolerant persister cells survive initial drug exposure through predominantly reversible, non-genetic adaptations, whereas stably resistant cells maintain reduced drug sensitivity after drug withdrawal and across subsequent cell divisions [50]. Longitudinal experiments comparing treatment-naive parental cells, drug-tolerant persister cells, and stably resistant cells could help distinguish treatment-induced adaptation from selection of pre-existing resistant populations, thereby clarifying whether chemotherapy induces the H3K14la–NEDD4–PTEN pathway or selects cells with pre-existing pathway activity. HDAC isoform-specific genetic perturbation and rescue experiments could further test the contribution of lactylation turnover [3,50,74,139]. Collectively, these studies implicate lactylation in distinct treatment-adaptive mechanisms, but their differences in therapeutic agents, substrates, and downstream pathways limit the generalization of a single lactylation-dependent resistance mechanism.

A complementary approach is to manipulate lactate production and transport using GNE-140, AZD3965, or genetic suppression of MCT4. Site-specific lactylation proteomics by LC-MS/MS, isotope tracing, and parallel acetylation profiling would help determine how these interventions affect the relevant molecular sites. Functional testing should compare chemotherapy alone, each intervention alone, and the corresponding combinations, supported by site-directed mutagenesis and genetic rescue [74,140,141]. Table 3 summarizes the corresponding questions, interventions, readouts, and interpretive limitations.

In mass spectrometry-based analyses, distinguishing lactyllysine from carboxyethyllysine requires particular attention because these modifications produce the same mass shift and cannot be reliably differentiated on the basis of mass alone [144]. Pharmacological interpretation also requires caution: GNE-140 has activity beyond LDHA, AZD3965 inhibits MCT1 with additional activity against MCT2, and the reported selectivity of RGFP966 for HDAC3 has not been confirmed by detailed kinetic analysis [133,140,141].

Causality therefore remains unresolved across the HDAC–lactylation axis. Individual models establish associations with resistance-related mechanisms, but chemotherapy-induced changes, selection of pre-existing resistant states, and the contributions of HDAC-dependent turnover have not been fully separated. Determining which enzymes control the relevant modifications is the next challenge, addressed in Section 7.2.

### 7.2. Defining Enzymatic Hierarchy: Which HDACs Are Dominant Delactylases In Vivo?

A second major challenge concerns the enzymatic landscape that regulates lactylation in vivo. Biochemical studies have identified HDAC1–3 as efficient histone delactylases in vitro, whereas cellular delactylase activity on histone substrates has been demonstrated most clearly for HDAC1 and HDAC3 [12,137]. HDAC2 has also been implicated in the regulation of non-histone lactylation involving METTL3, DHX15, and NUDT21 in specific cancer models [74,75,76]. However, the relative contributions of individual isoforms, their locus specificity, and their functional hierarchy in different tumor settings remain poorly resolved [12,137].

The picture is further complicated by evidence that several sirtuins also function as delactylases in specific cellular contexts. SIRT1 and SIRT3 have been identified as robust delactylases with distinct preferences for histone and non-histone substrates, and SIRT1-mediated delactylation has been linked to glycolytic regulation [77]. In gastric cancer, SIRT1 removes H3K18la and restrains a positive feedback loop involving H19, glycolysis, and histone lactylation [145]. SIRT6 has also been shown to remove histone lactylation at H3K9 and H3K18, with a substrate profile that partially differs from that of class I HDACs [78]. Furthermore, HDAC1–3 have been reported to catalyze lysine lactylation under defined biochemical conditions, although the cellular significance of this activity remains to be established [136]. These findings suggest that the enzymatic control of lactylation may be tissue-dependent, substrate-specific, and influenced by metabolic context [77,78,137,145].

A key priority for the field is therefore to define the in vivo hierarchy of HDAC and sirtuin delactylases, including how their activities differ across histone versus non-histone substrates, across genomic loci, and across therapy-naive versus resistant tumors. Pharmacological tools validated through isoform-focused genetic perturbation, together with biochemical activity measurements and site-resolved lactylation analysis, will be needed to establish these relationships. Without such resolution, it will remain difficult to determine which components of the delactylation machinery are most relevant for therapeutic intervention. The enzyme responsible for regulating a given lactylation site may therefore vary with substrate and cellular context. Whether the resulting modifications persist after therapy withdrawal is considered in Section 7.3.

### 7.3. Reversibility and Epigenetic Memory: Does Therapy Withdrawal Reset Lactylation Programs?

Because non-genetic therapy-adaptive states can be dynamic and reversible, an important question is whether lactylation-associated chromatin states are reset after therapy withdrawal or retained as a form of epigenetic memory [3,50]. Drug-tolerant persister states can partially revert following treatment cessation, indicating that at least some resistance-associated epigenetic programs are reversible [3,50,139]. However, it remains unknown whether lactylation marks are erased during this transition or persist long enough to influence the response to subsequent drug exposure [136,137,146]. This question is especially relevant to HDAC biology. If HDAC-mediated delactylation serves as a resetting mechanism, then alterations in HDAC activity during therapy withdrawal could determine whether lactylation-associated programs are erased or retained [12,137]. This raises the possibility that HDACs influence not only adaptation during treatment but also the persistence of lactylation-associated states during recovery.

During DNA replication, parental histones H3–H4 are redistributed to newly replicated DNA strands [147]. Established chromatin marks, including H3K9me3 and H3K27me3, can subsequently be maintained through read–write mechanisms that recognize existing modifications and promote their restoration [148]. Whether comparable mechanisms preserve histone lactylation patterns across cell divisions remains unclear, and, to our knowledge, their mitotic inheritance has not been directly demonstrated. Notably, prolonged H3K18 lactylation has been observed in monocytes/macrophages in the context of BCG-induced trained immunity, including persistence up to 90 days after vaccination [149]. However, persistence in this setting does not establish transmission of lactylation patterns to daughter cells. Given the influence of lactate availability and lactylation-associated enzymes [7,13,14], together with HDAC- and sirtuin-mediated delactylation [12,16,17], these patterns may instead be continuously re-established. Longitudinal profiling of locus-specific histone lactylation using validated antibodies and chromatin-based approaches, such as CUT&Tag or ChIP-seq, in synchronized or lineage-tracked cancer cells could help address this question. Combining these analyses with modulation of lactate availability or p300 activity, particularly following chemotherapy withdrawal, may help distinguish mitotic inheritance from ongoing enzymatic re-establishment.

Addressing this question will require longitudinal studies that measure lactylation before, during, and after treatment withdrawal, ideally using models that capture reversible drug-tolerant states rather than only fully established resistant clones [3,50,139]. Such studies could reveal whether lactylation marks function as transient stress signals or contribute to epigenetic memory in non-genetic resistance. Longitudinal drug-tolerant persister models incorporating treatment withdrawal and subsequent chemotherapy rechallenge, together with site-specific lactylation and HDAC activity measurements, could determine whether persistent lactylation changes predict or contribute to renewed drug tolerance [50,139]. Whether lactylation modifications are transient or retained remains unresolved. Understanding how persistent marks could influence cellular behavior also requires identifying the proteins that recognize them, as discussed in Section 7.4.

### 7.4. Functional Interpretation: Are There Bona Fide Lactylation Readers?

A defining property of regulatory histone marks is that they are not only written and erased but also interpreted by proteins that recognize them and convert them into downstream functional outputs. For lactylation, this interpretive layer remains only partially defined [137,138]. One of the clearest examples reported to date is DPF2, which has been shown to recognize lactylated histone tails, particularly H3K14la, and to promote transcriptional programs associated with tumorigenesis [58]. BRG1 (SMARCA4) has also been proposed as an H3K18la reader based on its preferential association with this mark during early iPSC reprogramming [150]. More recently, BRD9 was identified as an H3K18la reader in hepatocellular carcinoma. Recognition of H3K18la by the BRD9 bromodomain recruits the ncBAF complex to active promoters and enhancers, increases chromatin accessibility, and promotes oncogenic transcription [151].

The identification of these readers adds an interpretive layer to the HDAC–lactylation axis. Reader-focused experiments combining BRD9 genetic depletion and rescue with site-specific lactylation measurements could help determine whether reader recognition contributes to HDAC-dependent transcriptional responses. These studies should distinguish lactylation-dependent effects from the broader chromatin-regulatory functions of BRD9 [151]. By removing lactylation marks, HDACs could indirectly influence the recruitment of reader proteins and the transcriptional responses that follow, although this coupling has not yet been examined systematically [12,58,151]. Demonstrating such coupling would strengthen the view of lactylation as a regulated modification whose downstream effects can be tested experimentally. Future studies should therefore prioritize systematic identification and structural validation of lactylation readers, as well as functional assessment of how reader recognition differs between histone and non-histone lactylation. It will also be necessary to investigate whether HDAC-dependent changes in reader recruitment influence chemotherapy response, because the cited reader studies mainly address tumorigenesis or reprogramming [58,150,151].

### 7.5. Concluding Perspective

The central question of this review is whether HDAC-regulated lactylation causally influences chemotherapy resistance or sensitization. The current evidence supports three levels of interpretation. First, established biochemical findings show that lysine lactylation is enzymatically regulated, with p300 implicated in histone lactylation and HDAC1–3 exhibiting delactylase activity [7,12]. Second, studies examining lactylation of specific histone and non-histone proteins have reported contrasting relationships between lactylation and chemotherapy response [70,74,120]. However, the effects observed with entinostat cannot be attributed exclusively to altered lactylation because HDAC inhibition also affects acetylation [120]. Third, it remains unresolved whether chemotherapy directly induces lactate-dependent lactylation that causes resistance, whether HDAC-regulated marks persist after therapy withdrawal, and whether their recognition by reader proteins contributes to chemotherapy response.

Together, these findings identify HDAC-regulated lactylation as a candidate mechanism of chemotherapy resistance and sensitization whose importance depends on substrate and tumor context. A priority is to test whether validated HDAC isoform-focused perturbation, alone or combined with LDH or MCT1 inhibition or MCT4 suppression, alters chemotherapy sensitivity through defined lactylation sites. Site-specific lactylation proteomics by LC-MS/MS and genetic rescue experiments could establish mechanistic specificity and identify candidate response biomarkers. Longitudinal drug-withdrawal and rechallenge models, together with reader-focused perturbation, would clarify whether lactylation-associated states persist and produce functional consequences. At present, HDAC-regulated lactylation remains a promising but incompletely validated therapeutic mechanism; its relevance to treatment selection must be established through substrate-specific functional evidence.

## 8. Conclusions

The interplay between tumor metabolism and epigenetic regulation has emerged as an important framework for understanding cancer adaptation, with the HDAC–lactylation axis representing a key metabolic–epigenetic interface. Lactate functions not only as a metabolic byproduct but also as a signaling metabolite that can drive protein lactylation and influence transcriptional programs associated with tumor progression and therapeutic response [7,16,110]. Class I HDACs contribute to lactylation turnover by removing lactyl groups from histones and selected non-histone substrates, although their substrate specificity and functional effects vary across cellular contexts [12,74,75,76].

Together, this evidence places lactylation within a regulatory network linking metabolic state, histone-dependent transcription, and non-histone protein function [7,12,17,124]. The HDAC2–METTL3 pathway links non-histone delactylation to cisplatin resistance [74]. In contrast, H4K12la–GCLC has been associated with oxaliplatin resistance [70], whereas H3K18la-associated DHRS2 expression has been linked to cisplatin sensitization following entinostat treatment, although the effects of class I HDAC inhibition cannot be attributed exclusively to lactylation [120]. These findings indicate that the relationship between HDAC-regulated lactylation and chemotherapy response depends on the substrate and tumor context. Whether chemotherapy itself initiates the relevant lactylation changes, and how frequently these mechanisms operate across resistant tumors, remain unresolved.

From a therapeutic perspective, this framework highlights both opportunity and complexity. Because HDAC activity can influence lactylation dynamics in a context-dependent manner, the consequences of targeting this axis cannot be inferred from canonical HDAC biology alone [12,70,74,75,76,120]. Future studies integrating HDAC isoform-focused genetic and pharmacological perturbation (including HDAC3-biased inhibitors with experimentally verified selectivity), LDH/MCT-directed interventions, longitudinal drug-withdrawal and rechallenge models, site-specific lactylation proteomics by LC-MS/MS, and assessment of p300-dependent lactylation and reader-mediated responses with functional chemotherapy-response assays could identify substrate-specific mechanisms and candidate treatment-response biomarkers. Such evidence is needed before HDAC-regulated lactylation can be considered a validated therapeutic target for overcoming chemoresistance.

## Figures and Tables

**Table 1:** Representative histone lactylation-associated transcriptional regulation in cancer.

Histone Lactylation	Target Genes	Functional Context	Cancer Type
H3K18la	PPARD [67]	Metabolic adaptation under anaerobic/glycolytic stress	Breast cancer
	POM121 [9]	Oncogenic transcriptional circuitry with downstream MYC/PD-L1 activation	Non-small-cell lung cancer
	YBX1 [73]	Glycolysis-associated transcriptional feedback regulation	Hepatocellular carcinoma
H4K12la	GCLC [70]	Glutathione synthesis, ferroptosis suppression, and oxaliplatin resistance	Colorectal cancer stem cells
	RAD23A [72]	Super-enhancer-driven transcription linked to DNA damage tolerance	Ovarian cancer
H3K9la	RAD51, BRCA2 [71]	Homologous recombination repair and platinum resistance	Ovarian cancer

Abbreviations: H3K18la, histone H3 lysine 18 lactylation; H4K12la, histone H4 lysine 12 lactylation; H3K9la, histone H3 lysine 9 lactylation; PPARD, peroxisome proliferator-activated receptor delta; POM121, pore membrane protein 121; MYC, MYC proto-oncogene; PD-L1, programmed death-ligand 1; YBX1, Y-box binding protein 1; GCLC, glutamate-cysteine ligase catalytic subunit; RAD23A, RAD23 homolog A; RAD51, RAD51 recombinase; BRCA2, BRCA2 DNA repair associated; DNA, deoxyribonucleic acid.

**Table 3:** Proposed experimental approaches for investigating the causal contribution of HDAC-regulated lactylation to chemotherapy response.

Research Question	Experimental Approach	Principal Readout	Key Limitation
Does chemotherapy induce lactate-dependent lactylation?	Longitudinal treatment models, ^13^C-glucose/lactate tracing, site-specific LC-MS/MS	Lactate abundance and site-specific lactylation over time	Treatment induction versus selection; lactyllysine/CEL discrimination
Is lactate metabolism required for resistance?	LDH inhibition with GNE-140, MCT1 inhibition with AZD3965, MCT4 genetic suppression	Lactate flux, lactylation and chemotherapy sensitivity	LDH/MCT isoform overlap; metabolic compensation
Which HDAC regulates a specific lactylation site?	HDAC1/2/3 genetic perturbation and rescue; HDAC3-biased tools such as BRD3308 or RGFP966 with independent selectivity and target-engagement validation [142,143]	Site-specific lactylation, acetylation, and chemotherapy sensitivity	Imperfect inhibitor selectivity and overlapping HDAC functions
Does a lactylation event causally alter chemotherapy response?	Site-directed mutagenesis, genetic rescue, chemotherapy alone versus single interventions and combinations	Drug-response assays and substrate-specific functional effects	Mutations may alter protein function independently of lactylation

Abbreviations: LC-MS/MS, liquid chromatography–tandem mass spectrometry; CEL, Nε-(1-carboxyethyl)lysine; LDH, lactate dehydrogenase; MCT, monocarboxylate transporter; HDAC, histone deacetylase.

## Data Availability

No new data were created or analyzed in this study. Data sharing is not applicable to this article.
